# Moderating Effect of Burnout on the Relationship between Self-Efficacy and Job Performance among Psychiatric Nurses for COVID-19 in National Hospitals

**DOI:** 10.3390/medicina58020171

**Published:** 2022-01-24

**Authors:** Seongmi Lim, Youngok Song, Yoonyoung Nam, Youngmoon Lee, Duckjin Kim

**Affiliations:** 1 National Center for Mental Health, 127, Yongmasan-ro, Gwangjin-gu, Seoul 04933, Korea; lllmixmilll@gmail.com (S.L.); syonbmh@korea.kr (Y.S.); paulnam@korea.kr (Y.N.); humanishope@korea.kr (Y.L.); 2 Department of Nursing, Sungshin Women’s University, 55 Dobong-ro 76ga-ril, Gangbuk-gu, Seoul 01133, Korea

**Keywords:** psychiatric nursing, self-efficacy, burnout, job performance, COVID-19, nurses

## Abstract

*Background and Objective*: The unprecedented spread of infectious diseases, such as the COVID-19 pandemic, in psychiatric units has affected the self-efficacy, burnout, and job performances of psychiatric nurses. We conducted a survey to investigate the moderating effect of burnout on the relationship between the self-efficacy and job performances of psychiatric nurses. *Materials and Methods*: Validated and structured questionnaires were used to collect data from 186 nurses in psychiatric units for COVID-19. The data were analyzed using descriptive statistics, Pearson’s correlation coefficient, and a series of multiple linear regression analyses based on Baron and Kenny’s method using the SPSS 26.0 program. *Results*: Job performance was positively correlated with self-efficacy (r = 0.75, *p* < 0.001) but had no significant correlation with burnout (r = −0.11, *p* = 0.150). Self-efficacy was negatively correlated with burnout (r = −0.22, *p* = 0.002). Burnout among psychiatric nurses had significant moderating effects on self-efficacy and job performance (β = −0.11, *p* = 0.024). *Conclusions*: These findings indicate a need to prevent burnout and to enhance self-efficacy in psychiatric nurses to increase their job performances and serve as a basis for establishing strategies to deploy medical staff in the future.

## 1. Introduction

The COVID-19 pandemic caused by SARS-CoV-2 infections has led to a record-high fatality rate worldwide. The South Korean government raised the infectious disease alert level from blue to yellow on 20 January 2020 and officially activated its infectious disease response system. Although the daily number of confirmed cases remained low, including foreign entries, below 10 for a month, mass group infections began to surface from late February in a certain region and religious facility. Health authorities immediately enforced local regional closures and escalated disinfection efforts to block the routes of infection, and public health care institutions prepared negative-pressure facilities and isolation wards to house patients with confirmed infections [1]. During this time, approximately 100 inpatients in the psychiatry unit at Cheongdo Daenam Hospital tested positive for the virus and were placed in cohort isolation; however, because these long-term inpatients had severely weakened immune systems and low physical fitness, many of the patients eventually died from COVID-19 [2]. In response to this incident, nurses from the National Mental Health Center were sent to Cheongdo Daenam Hospital or were assigned to the care of COVID-19 patients transferred from the hospital. With the prolonged pandemic, there were unprecedented incidents in which the entire psychiatry ward was placed in cohort isolation and patients with severe mental illnesses, showing self-harm or aggressive behavioral problems, were transferred to negative-pressure isolation wards for COVID-19 care [3]. Treating these patients requires both medical intervention to battle the virus and psychiatric expertise to deal with symptoms of existing mental illnesses. Unfortunately, there were limited response measures, clinical guidelines, and evidence for nursing priorities pertaining to COVID-19 patients with mental disorders during the early days of the pandemic in February 2020 [4].

Ensuring adequate supply and efficient allocation of nursing professionals in each area of expertise is crucial and should be based on evidence to establish effective nursing strategies and systems amid mass infections such as COVID-19. In particular, considering that health disparities—limited health care access, including disinfection, diagnosis, and treatment, among certain socioeconomically and culturally vulnerable populations—in various parts of the world may exacerbate the prognosis of COVID-19, nursing professionals who are capable of understanding and properly responding to the unique clinical features of specific populations, such as the mentally ill population, are essential [5,6]. Several studies have analyzed the effects of stress, self-efficacy, and burnout among first-line health care providers working with mass group infections, namely nurses, on job performance and service delivery systems in Wuhan, China, and the United States [7,8,9]. One common complaint among nurses was the accumulating job stress due to their lack of knowledge about the COVID-19 disease and high workload from the surge of patients. They suggested collaborative systems and close communication with other professions and between senior and junior nurses as solutions to address this challenge. However, aside from a few qualitative studies on nurses’ experiences during the COVID-19 pandemic [10,11], studies on psychiatric nurses who provided care for COVID-19 patients are lacking in Korea.

Despite having inadequate knowledge about this infectious disease and the lack of clinical guidelines, nurses continue to provide care for patients while dealing with anxieties about the risk of contracting the infection and potentially spreading it to their families and with unresolved fatigue from the high workload [7,10,12]. The nurses are exposed to excessive tension and stress as they have to perform screening tests, to provide care for confirmed patients, and to practice infection control measures without adequate knowledge about the infection [3]. With the COVID-19 prolonged for nearly two years, nurses’ health is critically threatened, which may lead to burnout. Burnout refers to a state of physical, psychological, and emotional exhaustion as a result of excessive work or sustained pressure. Burnout reduces job satisfaction, subsequently hinders effectivity, and diminishes the quality of care [9].

The expectation and belief that one can take appropriate actions in a given situation is referred to as self-efficacy [13]. Nurses with low self-efficacy can suffer from stress and anxiety during difficulties, which in turn hamper work [8]. Numerous studies have found that high self-efficacy ensures proactive work and better goal achievement; it is an essential component in various conceptual frameworks created to understand how cognitive characteristics lead to actual behavior, such as the health belief model [14]. While self-efficacy is a predictor of various cognitive features, such as job stress, job performance, turnover intention, burnout, and their outcomes, the relationships between the factors vary depending on the data source and method of analysis [15]. A Korean study on nurses reported that self-efficacy had a positive effect on job performance [16,17].

As mentioned above, many studies have investigated nurses’ self-efficacy, burnout, and job performance, but none of the studies examined psychiatric nurses at national hospitals or those who provided care for patients with mental illnesses and infections with COVID-19. In Korea, there are currently six national mental health hospitals affiliated with the Ministry of Health and Welfare that function as base facilities that comprehensively provide mental health services for the community. It is speculated that nurses who work at a national hospital that features a characteristic vertical hierarchy prevalent in civil service organizations would have different experiences from nurses of other general hospitals due to their unique work environment and promotion systems. Hence, investigating the levels of self-efficacy, burnout, and job performance and their relationship among national hospital psychiatric nurses who provided care for patients with mental illnesses and infections with COVID-19 would be significant to generate foundational data for developing strategies that enhance their work quality.

Thus, this study aims to examine the levels of self-efficacy, burnout, and job performance and the moderating effect of burnout on the relationship between self-efficacy and job performance among psychiatric nurses who provided care for patients with mental illnesses and infections with COVID-19.

## 2. Materials and Methods

### 2.1. Study Design

This study is a cross-sectional descriptive observational study using a questionnaire.

### 2.2. Participants

Participants were conveniently sampled from nurses of six national hospitals (National Mental Health Center, Chuncheon National Hospital, Gongju National Hospital, Naju National Hospital, Bugok National Hospital, and Masan National Hospital) who had provided care for patients with mental illnesses and infections with COVID-19 in 2020. Among them, psychiatric nurses who voluntarily agreed to participate were extracted by convenience sampling.

The subjects of this study are nurses who had at least two weeks of experience caring for patients with confirmed or suspected COVID-19 infections among nurses working in a city national psychiatric hospital with an outbreak of COVID-19. Subjects were those who fully understood the purpose of the study and voluntarily agreed to participate in the study. Among the subjects, nurses who resigned or took a leave of absence at the time of data collection and nurses who belonged to other hospitals but were dispatched were excluded from the study subjects.

The descriptive survey was approved by the Institutional Review Board of National Center for Mental Health (IRB no. 116271-2020-35). Participants were given an information sheet explaining the purpose and method of the study, voluntary study participation and withdrawal, approximate duration of questionnaire completion, confidentiality of information, and lack of disadvantages from non-participation. Written consent was obtained from the participants.

The data were collected from 13 July to 3 August 2020 from consenting psychiatric nurses who had taken care of patients with mental illnesses and infections with COVID-19. Self-reporting questionnaires were sent and collected via mail or in person.

### 2.3. Instruments

A structured questionnaire was used to collect the data. The questionnaire consisted of 50 items, including 5 items for general characteristics, 17 items for self-efficacy, 21 items for burnout, and 7 items for job performance. Permission from the developers via email was obtained before using the instruments.

#### 2.3.1. Self-Efficacy

Self-efficacy was measured using the Self-efficacy Scale developed by Sherer et al. [18] and was modified and adapted for use on nurses by Chung [19]. This 17-item self-report uses a 5-point Likert scale ranging from 1 (strongly disagree) to 5 (strongly agree), with a higher score indicating higher self-efficacy. The reliability (Cronbach’s α) of the scale was 0.94 in the study by Chung [19] and 0.96 in this study.

#### 2.3.2. Burnout

Burnout was measured using the instrument developed by Pines et al. [20] and was modified and adapted for use on national hospital nurses by Lee [21]. This 21-item self-report uses a 5-point Likert scale ranging from 1 (strongly disagree) to 5 (strongly agree), with a higher score indicating greater burnout. The reliability (Cronbach’s α) of the scale was 0.85 in the study by Lee [21] and 0.79 in this study.

#### 2.3.3. Job Performance

Job performance is difficult to measure due to the broad scope of work in an organization, ambiguous criteria for distinguishing organizational- and individual-level performance, dependence of performance on the organization’s mission, and varying missions across organizations [22]. In this study, job performance was defined as one’s perceived success in performing their roles, and it was measured using the perceived job performance scale developed by Williams and Anderson [23] and translated by Lee [22]. This 7-item self-report uses a 5-point Likert scale ranging from 1 (strongly disagree) to 5 (strongly agree), with a higher score indicating higher perceived job performance. The reliability (Cronbach’s α) of the scale was 0.89 in the study by Lee [22] and 0.94 in this study.

### 2.4. Statistical Analysis

The collected data were analyzed using SPSS/WIN 26.0 (IBM Corp., Armonk, NY, USA). The participants’ general characteristics were analyzed using frequencies, percentages, means, and standard deviations. The levels of self-efficacy, burnout, and job performance were analyzed using mean and standard deviation. The differences in the major study parameters according to general characteristics were analyzed using an independent *t*-test and one-way ANOVA followed by Scheffe’s test. The correlations among self-efficacy, burnout, and job performance were analyzed using Pearson’s correlation analysis. The moderating effect of burnout on the relationship between self-efficacy and job performance was analyzed using hierarchical regression, as delineated by Baron and Kenny [24]. In step 1, the general characteristics that affect job performance were entered as the control variables, and in step 2, the independent variable (self-efficacy) was added. In step 3, the moderating variable (burnout) was added, and in step 4, the interaction variable (self-efficacy × burnout) was added to analyze the effects of these variables on the dependent variable (job performance). The moderating variable was centered to resolve the problem of multicollinearity between the independent and control variables.

## 3. Results

### 3.1. General Characteristics

Sex, age, marital status, and education level were analyzed as demographic characteristics. The participants comprised 85.5% women and 14.5% men. Age was divided into 20–29 years (11.3%), 30–39 years (32.8%), 40–49 years (17.7%), and ≥50 years (38.2%). Regarding marital status, 26.3% were single and 73.7% were married. Regarding education, 10.8% had an associate degree, 53.8% had a bachelor’s degree, and 35.5% had a master’s degree or higher. Length of career in psychiatric nursing was examined as a job-related characteristic. The length of career was divided into 1–9 years (42.5%), 10–19 years (14.5%), and ≥20 years (43%) (Table 1).

The mean self-efficacy was 3.84 ± 0.58 out of a possible score of 2–5. The mean burnout score was 3.10 ± 1.18 out of a possible score of 1–5, and the mean job performance score was 4.04 ± 0.55 out of a possible score of 3–5 (Table 2).

### 3.2. Differences in Self Efficacy, Burnout, and Job Performance According to General Characteristics

Self-efficacy differed significantly according to age (F = 9.43, *p* < 0.001), marital status (t = −2.40, *p* = 0.17), education level (F = 7.55, *p* > 0.001), and length of psychiatric nursing career (F = 13.14, *p* < 0.001). In terms of age, nurses aged ≥ 50 years had a higher score (4.08 ± 0.57) than nurses aged 30–39 years (3.57 ± 0.56). Married nurses (3.9 ± 0.59) scored significantly higher than single nurses (3.67 ± 0.55), but the difference between the groups was not statistically significant in the post hoc analysis. In terms of education, nurses with a master’s degree or higher (4.30 ± 0.54) had a significantly higher self-efficacy score than those with a bachelor’s degree (3.69 ± 0.59). In terms of length of psychiatric nursing career, nurses with a career of ≥20 years (4.07 ± 0.56) had a significantly higher self-efficacy score than nurses with a career of 1–9 years (3.62 ± 0.54) (Table 1). Burnout did not differ significantly according to the general characteristics.

Job performance significantly differed according to age (F = 16.25, *p* < 0.001), marital status (t = −3.06, *p* = 0.003), education level (F = 9.01, *p* < 0.001), and length of psychiatric nursing career (F = 29.91, *p* < 0.001). The job performance scores for different age groups were 4.35 ± 0.53 for ≥50 years, 3.97 ± 0.49 for 40–49 years, 3.77 ± 0.43 for 30–39 years, and 3.86 ± 0.56 for 20–29 years. Married nurses had significantly better job performances (4.11 ± 0.54) than single nurses (3.83 ± 0.54), and nurses with a master’s degree or higher (4.25 ± 0.56) had significantly better job performances than nurses with a bachelor’s degree (3.89 ± 0.53), consistent with the self-efficacy scores. Job performance also significantly differed according to length of psychiatric nursing career, where those with a career of ≥20 years had the highest score (4.33 ± 0.52), followed by 10–19 years (4.03 ± 0.53) and 1–9 years (3.74 ± 0.42) (Table 1).

### 3.3. Correlations among Self-Efficacy, Burnout, and Job Performance

Job performance was positively correlated with self-efficacy (r = 0.75, *p* < 0.001) but not significantly correlated with burnout (r = −0.11, *p* = 0.150). Self-efficacy was negatively correlated with burnout (r = −0.22, *p* = 0.002) (Table 3).

### 3.4. Moderating Effect of Burnout on the Relationship between Self-Efficacy and Job Performance

The moderating effect of burnout on the relationship between self-efficacy and job performance was analyzed in accordance with the hierarchical regression method proposed by Baron and Kenny [24]. The correlations among the independent variables were analyzed to review the basic assumptions for regression, and the correlation coefficients ranged from 0.29 to 0.75. Thus, multicollinearity was analyzed; tolerance was under 1.0, with a range of 0.22–0.98; and variance inflation factor was under 10, with a range of 1.06–4.63, confirming the absence of multicollinearity. Next, the assumptions of residuals were examined, and the assumptions of normality, homoscedasticity, and linearity were met. In the autocorrelation test, the Durbin Watson statistic was close to 2 (2.19), confirming the absence of autocorrelation. The final moderating effect was analyzed using hierarchical regression, and the results are presented in Table 4.

**Figure 1:**
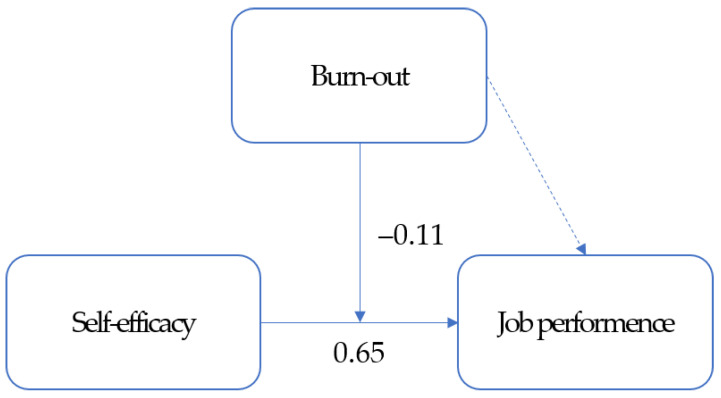
Moderating effect of burnout on the relationship between self-efficacy and job performance.

In step 1, age, marital status, education level, and psychiatric nursing career, which significantly differed in relation to job performance, were entered as control variables. Marital status and education level were categorical variables, so they were dummy coded. Model 1, which only included control variables, explained 26.8% of the variance in job performance (F = 13.18, *p* < 0.001). Psychiatric nursing career (β = 0.39, *p* = 0.006) was identified as a significant predictor. In step 2, the independent variable (self-efficacy) was added. Model 2 explained 62.5% of the variance in job performance, 35.7% higher than that of Model 1 (F = 170.11, *p* < 0.001). The control variables psychiatric nursing career (β = 0.22, *p* = 0.001) and independent variable self-efficacy (β = 0.65, *p* < 0.001) were identified as significant predictors of job performance. In step 3, the moderating variable burnout was added. Model 3 did not explain the variance in job performance (F = 1.83, *p* = 0.178). In step 4, self-efficacy and burnout interactions were added. Model 4 explained 63.9% of the variance in job performance (F = 5.19, *p* = 0.024). The R square increased over the models, from 62.5% in Model 2 to 62.8% in Model 3. Although the moderating variable in step 3 was not statistically significant, the interaction term was statistically significant in step 4, confirming that burnout was a pure moderator variable [25]. In other words, burnout does not statistically affect job performance, but it has a moderating effect that reduces job performance by interacting with self-efficacy. The control variables psychiatric nursing career (β = 0.22, *p* = 0.001), independent variable self-efficacy (β = 0.69, *p* < 0.001), and self-efficacy and burnout interaction (β = −0.11, *p* = 0.024) significantly affected job performance (Figure 1).

## 4. Discussion

This study investigated the levels of self-efficacy, burnout, and job performance in psychiatric nurses; the differences in self-efficacy, burnout, and job performance according to the general characteristics of psychiatric nurses; the correlations among self-efficacy, burnout, and job performance in psychiatric nurses; and the moderating effect of burnout on the relationship between self-efficacy and job performance in psychiatric nurses.

The mean self-efficacy score in this study was 3.84 out of 5, which is higher than the 3.56 among nurses of university hospitals or higher-level hospitals in the study by Chung [19]. In terms of general characteristics, nurses aged 50 years or older, married nurses, nurses with a master’s degree or higher, and nurses with a psychiatric nursing career of 20 years or longer showed high self-efficacy. The participants were veteran nurses with rich experience in demographics of work-related characteristics (38.2% were ≥50 years of age, and 43.0% had ≥20 years of experience in their psychiatric nursing careers). Nurses working in psychiatric units at national hospitals amidst the fear of COVID-19 participated in our study. Hence, these nurses have a higher level of confidence and competence to successfully perform a given task compared with nurses in other hospitals. However, there are no available data on the details of how these nurses were placed in psychiatric units for COVID-19 and their decision-making. Additionally, due to frequent changes in the work standards and guidelines for psychiatric nurses and line of command during the surge of COVID-19 in the community, there are limitations in reviewing these results based on additional analysis or the literature according to nurses’ general characteristics.

The mean burnout score was 3.10 out of 5. Considering that psychiatric nurses reported a lower burnout score (2.45) compared with other ward nurses in the study by Lee and Kim [26], we can speculate that our participants had a higher burnout score compared with before the pandemic. As workers of national hospitals that play a pivotal role in national anti-infection measures, they would have been heavily burdened with the COVID-19 care of patients with mental illnesses and the anti-infection measures during the early days of the pandemic, when there was an explosive surge in the number of patients.

Burnout did not statistically differ according to the general characteristics in our study, which is in contrast with previous findings that burnout decreases with increasing age, job position, and career [26]. In this study, the mean burnout score according to each general characteristic was 3 or higher, which may be attributable to the burden of interpreting infection management guidelines and making decisions at every moment during the early days of the pandemic, throughout which an effective infection management system was lacking. Even older nurses in higher positions and with longer careers could have experienced a greater burden of work due to the nature of national hospitals that serve as the control tower for national anti-infection measures. Therefore, we can observe that psychiatric nurses in national mental health hospitals experienced severe burnout regardless of age, job position, and career during the pandemic, highlighting the need for governmental and social support to prevent burnout during future pandemics.

The mean job performance score was 4.04, which was higher than that reported by Lee [22] among franchise workers (3.90) using the same instruments and that reported by Oh and Wee [17] among nurses of public hospitals outside of Seoul’s metropolitan region (3.79). In terms of general characteristics, nurses aged 50 years or older, married nurses, nurses with a master’s degree or higher, and nurses with a longer psychiatric nursing career had higher job performance scores. This is very similar to the higher job performance with increasing age, length of career, and married nurses in the study by Oh and Wee [17]. As previously mentioned, most of our participants were veteran nurses with rich experience, and for this reason, they were confident in their work and perceived themselves to be successfully performing their roles. In addition, psychiatric hospitals feature a positive, interpersonal relationship-oriented culture that emphasizes calling, trust, and cooperation among the members of the organization. These cultural aspects lead to high job satisfaction and organizational commitment, thereby promoting positive organizational performance [27]. Particularly, the high job performance among nurses of national mental health hospitals compared with their counterparts in private hospitals is similar to our results showing high job performance among our participants [27]. Hence, it is important to pay attention to personnel welfare, such as career management and reward systems to promote long-term retention of nurses, to improve job performance among psychiatric nurses in national hospitals, and to boost organizational performance. Furthermore, in addition to individuals’ perceived job performance, systematic studies that assess job performance at the organizational level are needed.

Regarding the correlation between self-efficacy and burnout, we found a negative correlation between the two among our participants. Previous studies also reported a negative correlation between self-efficacy and burnout among nurses [15]. Protective factors against burnout are divided into environmental factors and individual factors, and self-efficacy is a modifiable individual cognitive factor [15], thus calling for strategies that will improve self-efficacy in psychiatric nurses to lower their burnout. Particularly, programs that prevent burnout and improve self-efficacy should be developed and administered to psychiatric nurses amid the rising prevalence of burnout during the COVID-19 pandemic.

We observed a strong positive correlation between self-efficacy and job performance, akin to previous studies that also reported a positive correlation between the two factors in nurses [16,17], suggesting that job performance increases with increasing self-efficacy. Since there is a two-way relationship between nurses’ self-efficacy and job performance, an interactive upward strategy is needed to improve job performance through self-efficacy and self-efficacy through positive feedback on job performance [16].

In this study, burnout and job performance were not significantly correlated. This was partially in line with the results of Kim et al. [28], where burnout and job performance were not significantly correlated among nurses in comprehensive nursing care service wards but were significantly correlated in nurses of general wards. We can speculate that nurses who work in specialty wards or national mental health hospitals that are equipped with a stable system may experience high burnout, but it may not directly affect their job performance because of the high work efficiency in these environments. However, one limitation of our study is that, due to the lack of studies that used the job performance scale on nurses as we did, we compared our results with previous studies that examined a similar concept known as nursing work performance.

Burnout was found to have a moderating effect on the relationship between self-efficacy and job performance. In other words, burnout does not directly affect job performance but moderates the effect of self-efficacy in diminishing job performance. Although we cannot directly compare our findings with those of the literature due to a lack of studies that examine the moderating effect of burnout on the relationship between self-efficacy and job performance, our results are in line with those reported by Yang et al. [29], where emotional exhaustion moderates the relationship between servant leadership and job performance. People with high self-efficacy set challenging goals, invest much effort into achieving their goals, and strive to accomplish their tasks, all of which positively influence their job performances [15,16]. Thus, psychiatric nurses with high self-efficacy display good job performances, but if their burnout increases in special circumstances such as the COVID-19 pandemic, it may interact with their self-efficacy and lower job performance. Due to the prolongation of the COVID-19 pandemic, psychiatric nurses at national hospitals are required to provide extended care for patients with mental disorders and COVID-19, and this would naturally lead to greater burnout compared with in other nurses. As burnout negatively influences self-efficacy and thus lowers job performance, administrative and institutional support to ensure adequate rest and rewards are needed to prevent burnout in psychiatric nurses.

Essentially, there was a significant negative correlation between burnout and self-efficacy, and the interaction between burnout and self-efficacy significantly influenced job performance. Strategies to alleviate burnout among national hospital nurses include reducing job stress, respecting autonomous decision-making at work, and increasing workers’ self-efficacy [21]. A previous study reported that health care providers in Wuhan displayed a lower level of burnout during the COVID-19 pandemic compared with that before the pandemic and suggested that factors such as considerate management by health care institutions and the elevated social reputation of health care providers have contributed to the result [30]. Based on these results, health care institutions and the government should implement more considerate management such that psychiatric nurses working in special environments, such as national mental health hospitals, would not experience burnout. In particular, considering their work environment, which calls for concurrent mental health care and COVID-19 care, new personnel need to be added and new material infrastructures and systems need to be established.

This is the first study to investigate self-efficacy, burnout, and job performance among the psychiatric nurses of national hospitals who provide care for patients with mental illnesses and infected with COVID-19. Furthermore, it confirms that burnout moderates the relationship between self-efficacy and job performance in psychiatric nurses. Moreover, our findings that psychiatric nurses’ burnout in special circumstances, such as the COVID-19 pandemic, interacts with their self-efficacy to influence job performance, serves as useful foundational data for developing strategies to improve job performance.

This study has several limitations. First, we used instruments that were developed in foreign countries and adapted into Korean, which poses limitations in performing multilateral analyses of the context, perception, and attitudes pertaining to self-efficacy, burnout, and job performance in Korean psychiatric nurses during the COVID-19 pandemic. Second, the participants were convenience-sampled, so generalizing the findings to the entire psychiatric nurse population requires caution.

We present the following suggestions for subsequent studies. First, studies should verify the relationship between self-efficacy, burnout, and job performance found in this study. Second, future research should employ instruments that can measure job performance at the organizational level in addition to at the individual level used in this study. Finally, additional studies are needed to explore the various predictors of job performance among psychiatric nurses.

## 5. Conclusions

This study aimed to investigate the moderating effect of burnout on the relationship between self-efficacy and job performance among nurses in psychiatric units of six national hospitals for COVID-19. The results confirmed that burnout affects job performance by interacting with self-efficacy. One key significance of this study is that it presents foundational data for devising strategies to improve the job performances of psychiatric nurses. Based on our results, strategies that prevent burnout and improve self-efficacy should be developed and implemented for psychiatric nurses in national hospitals to improve their job performances.

## Figures and Tables

**Table 1:** Differences in self-efficacy, burnout, and job performance according to general characteristics (*n* = 186).

Characteristics	Categories	*n* (%)	Self-Efficacy		Burnout		Job Performance	
			M ± SD	t or F (*p*) Scheffé	M ± SD	t or F (*p*) Scheffé	M ± SD	t or F (*p*) Scheffé
Sex	Female	159 (85.5)	3.86 ± 0.60	0.93 (0.353)	3.12 ± 0.43	1.47 (0.144)	4.06 ± 0.57	1.40 (0.165)
	Male	27 (14.5)	3.74 ± 0.47		2.98 ± 0.47		3.9 ± 0.45	
Age	20~29 ^a^	21 (11.3)	3.79 ± 0.53	9.43 (<0.001) b < d	3.24 ± 0.4	0.93 (0.429)	3.86 ± 0.56	16.25 (<0.001) b,a,c < d
	30~39 ^b^	61 (32.8)	3.57 ± 0.56		3.1 ± 0.48		3.77 ± 0.43	
	40~49 ^c^	33 (17.7)	3.86 ± 0.49		3.08 ± 0.38		3.97 ± 0.49	
	≥50 ^d^	71 (38.2)	4.08 ± 0.57		3.06 ± 0.45		4.35 ± 0.53	
Marital status	Unmarried	49 (26.3)	3.67 ± 0.55	−2.40 (0.017)	3.1 ± 0.46	−0.048 (0.962)	3.83 ± 0.54	−3.06 (0.003)
	Married	137 (73.7)	3.9 ± 0.59		3.1 ± 0.43		4.11 ± 0.54	
Education	Associate degree ^e^	20 (10.8)	3.93 ± 0.48	7.55 (0.001) f < g	3.09 ± 0.47	2.58 (0.780)	4.05 ± 0.41	9.01 (<0.001) f < g
	Bachelor’s degree ^f^	100 (53.8)	3.69 ± 0.59		3.16 ± 0.45		3.89 ± 0.53	
	≥Graduate school ^g^	66 (35.5)	4.03 ± 0.54		3.01 ± 0.4		4.25 ± 0.56	
Career of psychiatric nursing	1~9 ^h^	79 (42.5)	3.62 ± 0.54	13.14 (<0.001) h < j	3.13 ± 0.46	0.32 (0.726)	3.74 ± 0.42	29.91 (<0.001) h < i < j
	10~19 ^i^	27 (14.5)	3.81 ± 0.55		3.07 ± 0.42		4.03 ± 0.53	
	≥20 ^j^	80 (43)	4.07 ± 0.56		3.08 ± 0.43		4.33 ± 0.52	

Letters that are not superscripted represent the content before the corresponding superscripted letters.

**Table 2:** Mean scores for self-efficacy, burnout, and job performance (*n* = 186).

Variable	M ± SD	Range
Self-efficacy	3.84 ± 0.58	2~5
Burnout	3.10 ± 1.18	1~5
Job performance	4.04 ± 0.55	3~5

**Table 3:** Correlation among self-efficacy, burnout, and job performance (*n* = 186).

Variable	Self-Efficacy	Burnout	Job Performance
	r (*p*)	r (*p*)	r (*p*)
Self-efficacy	1		
Burnout	−0.22 (0.002)	1	
Job performance	0.75 (<0.001)	−0.11 (0.150)	1

**Table 4:** Moderating effect of burnout on the relationship between self-efficacy and job performance (*n* = 186).

Variables	Categories	Model 1			Model 2			Model 3			Model 4		
		β	t	*p*	β	t	*p*	β	t	*p*	β	t	*p*
Controlling variables			−3.66	0.000		−3.24	0.001		−3.21	0.002		−3.37	0.001
Age		0.08	0.86	0.391	0.04	0.63	0.527	0.05	0.72	0.470	0.05	0.71	0.481
Marital status †	Married	0.03	0.49	0.624	0.01	0.20	0.840	0.01	0.12	0.906	0.01	0.11	0.917
Education ‡	Bachelor’s degree	−0.13	−1.21	0.229	−0.01	−0.08	0.940	−0.01	−0.11	0.910	−0.01	−0.06	0.951
	≥Graduate school	−0.01	−0.08	0.937	0.01	0.15	0.878	0.02	0.23	0.821	0.01	0.18	0.857
Career of psychiatric nursing		0.39	4.12	0.000	0.22	3.27	0.001	0.22	3.16	0.002	0.22	3.24	0.001
Self-efficacy					0.65	13.04	0.000	0.66	13.08	0.000	0.69	13.42	0.000
Burnout								0.06	1.35	0.178	0.06	1.24	0.216
Self-efficacy × Burnout											−0.11	−2.28	0.024
F(*p*)		13.18 (<0.001)			170.11 (<0.001)			1.83 (0.178)			5.19 (0.024)		
R^2^		0.27			0.63			0.63			0.64		
Change of R^2^		0.25			0.61			0.61			0.62		
adj. R^2^		0.27			0.36			0.00			0.01		

Reference group: † Unmarried, ‡ Associate degree.

## Data Availability

The data presented in this study are available from the corresponding author upon request due to privacy restrictions.
